# A Preliminary Compilation of a Digital Video Library on Triggering Autonomous Sensory Meridian Response (ASMR): A Trial Among 807 Chinese College Students

**DOI:** 10.3389/fpsyg.2019.02274

**Published:** 2019-10-15

**Authors:** Mengjie Liu, Qiang Zhou

**Affiliations:** Department of Psychology, Wenzhou Medical University, Wenzhou, China

**Keywords:** autonomous sensory meridian response, material library, trigger, intensity of tingles, frequency of tingles

## Abstract

Autonomous Sensory Meridian Response (ASMR) is a type of tingling, static-like sensation that is triggered by special audiovisual stimulation. The sensation passes through the scalp and the back of the neck, sometimes even spreading to the ends of the extremities. In recent years, research on ASMR has been gradually increasing. However, few collections of ASMR video material have been evaluated so far. In the present study, 807 Chinese participants (ASMR participants = 435, non-ASMR participants = 372) were asked to evaluate two types of ASMR videos and one control video, assessing the intensity and duration of tingling sensations triggered by these videos. A total of 60 ASMR videos were screened. The subjective assessment of the experimental group on ASMR intensity and duration, as triggered by the ASMR video material, demonstrated that the library contains 60 ASMR videos that can effectively trigger ASMR in participants who are able to experience ASMR. This video library was then subjected to a test which revealed that Cronbach’s α = 0.933. This proves that the library has good reliability, that it can effectively trigger ASMR in participants who are able to experience ASMR, and that it can be used as experimental material in future ASMR research.

## Introduction

“*What ASMR evokes is the ability to pay attention to daily noise production and create an emotional response for the audience: these sounds are intimate because the listener has to be close enough to hear them*.” (Smith and Snider, 2019)

In recent years, ASMR videos, a gimmicky type of video that induces relaxation and promotes sleep, has become popular on the internet. Its related communities continue to grow and develop. Autonomous Sensory Meridian Response (ASMR) is a sensory phenomenon in which individuals, in response to specific triggering audio and visual stimuli, experience a tingling, static-like sensation across the scalp and the back of the neck, sometimes spreading to further areas (Barratt and Davis, 2015).

Many scientists have focused their attention on ASMR triggers, conducting research on their characteristics. They found that while there are great differences in ASMR triggers among individuals (Barratt and Davis, 2015), participants prefer pleasant, attractive, relaxing, and non-hazardous ASMR videos (Barratt et al., 2017). Researchers believe that one of the reasons people enjoy watching ASMR videos may be their “therapeutic utility,” that is, ASMR videos are often used as a tool for relaxation and a coping strategy against anxiety (Barratt and Davis, 2015).

However, It was not until 2018 that a physiological correlate of ASMR was measured. Experiment results indicated that ASMR is a pleasant, calm, but activatable sensory experience unique to people who can experience it (Poerio et al., 2018). In addition, ASMR-related brain studies found that there was significantly increased activation in specific areas of the brain during moments of ASMR “tingling” compared to non-tingling moments. The brain regions found most active during the tingling sensations were the nucleus accumbens, medial prefrontal cortex, insula and secondary somatosensory cortex. These are the first results to demonstrate a unique neural activation associated with the experience of ASMR (Lochte et al., 2018).

Although ASMR research has become increasingly in-depth, there is a lack of research into ASMR videos. Videos are among the few materials currently available for ASMR research, as ASMR shows strong individual differences. Besides video material, only the ASMR Checklist compiled by Beverley et al. (2017) is available, although it is limited to 14 videos. Therefore, this study intends to develop a systematic and comprehensive ASMR-triggering digital video library. The aim is to build a database for future research on topics such as trigger mechanisms and the potential therapeutic functions of ASMR.

## Materials and Methods

### Participants

The experiment was approved by the Ethics Committee of the Department of Psychology of Wenzhou Medical University. The participants were randomly recruited undergraduate students at Wenzhou Medical University. A total of 821 participants were gathered. The experiment was conducted in a quiet laboratory. Each time approximately 30 participants sat in front of a computer with the same configuration for about half an hour (HP ProOne 400G3 20.0-in Non-Touch AiO), 0.5 m from the screen, wearing the same headset (Rovers K800). Due to, among other things, errors in the software used in the experiment, 14 participants had to be excluded, so 807 samples were retained (*N* = 807, 75.1% were female, *M*_*age*_ = 19.4 years, *SD* = 1.108, Range = [18, 24]). The detailed results are shown in Table 1.

**TABLE 1:** Demographic variable information.

	**The participants type**								
	**ASMR**			**Non-ASMR**			***t***	***df***	***p***
**Gender**	***N***	**Age(year)**		***N***	**Age(year)**				
		***M***	***SD***		***M***	***SD***			
Female	311	19.47	1.19	295	19.46	1.07			
Male	124	19.35	1.01	77	19.00	1.01			
Total	435	19.43	1.14	372	19.37	1.07	0.880	805	0.379

*This table describes the age and gender differences of subjects in different groups (ASMR group and Non-ASMR group). The results showed that there was no age difference between the ASMR group and Non-ASMR group on the gender.*

### Stimuli and Procedure

There is a great number of AMSR videos published on YouTube. We initially shortlisted 100 videos. Based on the YouTube channels’ number of subscribers, the number of video playbacks, the community discussions, and the types of ASMR trigger, we selected 60 out of these first 100 (see Supplementary Appendixes A,B for details on the material). All videos were used with consent from the creators. Each video was clipped to a 25-frame per second video in MPEG (640 × 480) using Power Director 15 (see Supplementary Appendix C for thumbnails of the material). The length of each video varied from 1–3 min. The following videos were included in this study: (a) four control videos; (b) 30 ASMR-triggering videos with semantic dialogues (half of them performed by men and half by women); (c) 30 ASMR-triggering videos without semantic dialogues (half of them performed by men and half by women). Videos with semantic dialogues refer to those in which social interaction dialogues occur, and videos without semantic dialogues refer to those that only include ASMR triggers and do not include social interaction dialogues. Each ASMR video contained one or more triggers, for example, one video included semantic dialogue, whispering, sounds of ear massage and sounds of hair combing. The control group videos were also selected from YouTube. It contained similar content to the ASMR videos, including close-up shots and slow, repeated motions, but did not include ASMR triggers. These mimicked the content of ASMR videos as closely as possible (e.g., spoken instructive and demonstration videos with actors facing the viewers directly, and soundonly videos with the camera focused on a close-up scene). However, they did not contain ASMR triggers and were not deemed to be potentially ASMR-inducing (Poerio et al., 2018). Whether video contains ASMR ingredients is defined according to the study of “the effective characteristics of ASMR triggers” in the “understanding the triggers”. For instance, “video content should be natural (no scripting); Connect with the audience; The voice should be natural and low pitched; In video background music should not be used and so on” (Barratt et al., 2017). The control video we selected did not contain any of these components, so we defined it as control video (Supplementary Datasheet S1 and S2).

The experiment was programed in PsychoPy 3.0.6 (Peirce et al., 2019). The brightness and contrast of the display were uniformly set at 50%. Group testing was employed among the participants. After the participants provided their demographic information, they watched one video from each category. Videos were selected at random from video materials prepared in advance and presented at random. After watching each video, participants were asked to assess the tingling sensations felt while watching the video and their frequency. Due to the individual differences of subjects, some subjects’ ASMR experience may be intermittent, but the duration of their ASMR experience lasts for a short time, it will greatly destroy the subject’s ASMR experience.

**FIGURE 1:**
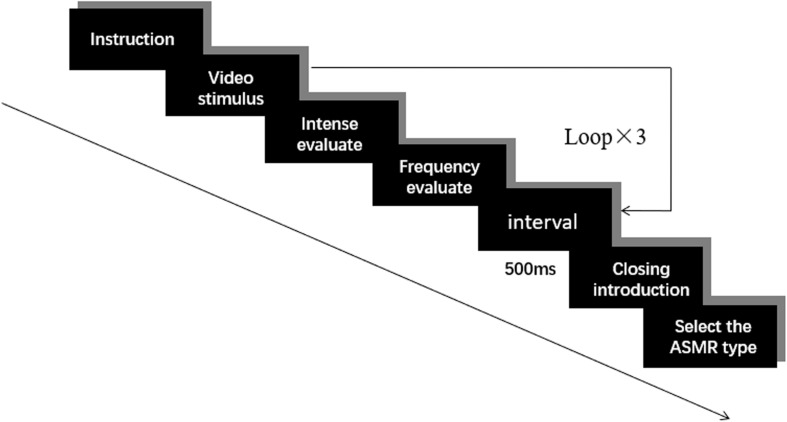
Experimental design. This figure describes the steps of the experiment. In the experimental instructions, the participants needed to watch three videos from different categories randomly. After watching each video, they were asked to rate the intense and frequency of ASMR that they felt. Subsequent videos were presented at intervals of 500 ms. Then the concept of ASMR will be explained. Group testing was employed among the participants.

Scores were based on their immediate feeling and participants were requested to avoid spending too much time thinking. Subsequent videos were presented at intervals of 500 ms. The experiment flow chart is shown in Figure 1. After watching the videos, participants were briefed on the definition of ASMR and provided a response on whether they could experience ASMR. Participants in the ASMR group were selected as follows: After the participants watched all the videos and understood the relevant definitions of ASMR, they were asked “Based on your life experience and the videos you just watched, do you think you can experience ASMR?” Participants who chose “Yes” were classified as the ASMR experimental group. Those who chose “No” were classified as the control group.

## Results

### Two-Dimensional Score Analysis of ASMR

The intensity of arousal of ASMR experience by videos in the library was measured for each video through the self-reported ASMR intensity measurement. Participants were required to score each video on a 9-point scale (0–8) after watching it. During the final result analysis, some participants (*N* = 26) reported being “unsure” about the intensity of the video, indicating that they had difficulty judging ASMR intensity triggered by the stimulus. In cases where the corresponding frequency was not zero, the average intensity reported by participants in the group (experimental and control group) for this specific video would be assigned as the value. In all other cases, the value assigned was zero. Participant reports on the intensity and the frequency of the tingling sensation triggered by the videos are shown in Supplementary Appendix C.

Spearman correlation analysis was performed on ASMR two-dimensional scores given for the ASMR videos by participants in the experimental group. The intensity of arousal and frequency of sustained sensation were significantly correlated (*r* = 0.913, df = 433, *p* < 0.01).

**FIGURE 2:**
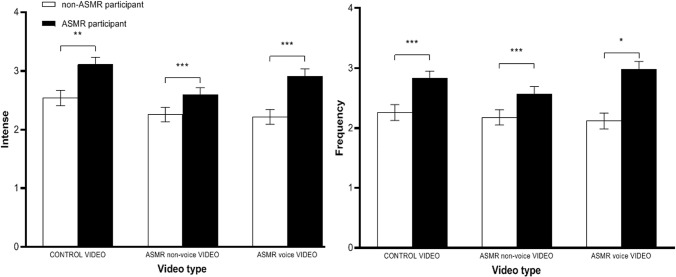
Differences between ASMR group on self-reported tingle from viewing different videos. This figure describes the differences between ASMR group on self-reported tingles from viewing different videos. The results show that the intensity of arousal and the frequency of persistent sensations reported by the participants in the ASMR group were significantly higher than those in the non-ASMR group. The intensity and duration of arousal reported by the non-ASMR group did not differ among the three groups of videos. ^∗^*p* < 0.05, ^∗∗^*p* < 0.01, ^∗∗∗^*p* < 0.001.

The evaluation of intensity was carried out among both groups (ASMR participant and non-ASMR participant) for three types of videos (control videos, ASMR videos with semantic dialogues, ASMR videos without semantic dialogues) through ANOVA with a hybrid design and repeated measurements. The results show that the main effect on participant types was significant (*F* (1,805) = 16.584, *p* < 0.01, η^2^*p* = 0.020). Compared with the control group, the participants in the experimental group had more intense feeling about the control videos (M_*diff*_ = 0.573 [0.216, 0.931], *p* = 0.002), ASMR videos without semantic dialogues (M_*diff*_ = 0.668 [0.324, 1.013], *p* < 0.01), and ASMR videos with semantic dialogues (M_*diff*_ = 0.351 [0.010, 0.693], *p* < 0.01). The main effect of video type was significant (*F* (2,804) = 6.781, *p* = 0.001, η^2^*p* = 0.017). The intensity of the tingling, static-like sensation induced by control videos was stronger than that induced by ASMR videos with semantic dialogues (M_*diff*_ = 0.404 [0.138, 0.670], *p* = 0.001), and the tingling and static-like sensation induced by control videos was stronger than that induced by ASMR videos without semantic dialogues (M_*diff*_ = 0.261 [0.017, 0.505], *p* = 0.032), as shown in Figure 2.

ANOVA of repeated measurements revealed that the main effect of participant type was significant (*F* (_1_, _805_) = 73.102, *p* < 0.01, η^2^*p* = 0.24). The experimental group experienced more frequent tingling sensations for control videos (M_*diff*_ = 0.571 [0.220, 0.923], *p* = 0.001), ASMR videos without semantic dialogues (M_*diff*_ = 0.862 [0.497, 1.227], *p* < 0.01) and ASMR videos with semantic dialogues (M_*diff*_ = 0.378) [0.028, 0.729], *p* = 0.035) than the control group. No other effects were significant.

Chi–square test was performed on gender given for videos of different type by participants in the experimental group. After watching the non-voice VIDEO of ASMR, the ASMR intensity of female in ASMR participant is significantly stronger than that of male in ASMR participant, *t* (380) = 1.982, *p* = 0.048. The ASMR intensity of female in ASMR participant after watching the ASMR VOICE VIDEO is significantly stronger than that of male in ASMR participant, *t* (360) = 1.959, *p* = 0.051. The ASMR intensity of female in ASMR participant after watching the control video is not significant compared with that of male in ASMR participant, *t* (370) = −0.033, *p* = 0.974.

### Internal Consistency Reliability of the ASMR Video Material Library

Considering that, in this experiment and in future experiments, participants were not and will not be able to assess all stimuli (the evaluation of all videos took about 3 h), the average intensity and frequency of the 60 stimuli were arranged in order of strong to weak intensity, thus strong, medium and weak. For greater convenience of use in future research, the first recommended ASMR digital video library includes the following: four videos with the highest average intensity and frequency (“ear licking,” “eating caviar and sea grape,” “electronic cigarettes,” “grind salt”); four videos with medium triggering intensity (“archeological dig,” “roleplay of energy healing,” “whispering and personal attention,” “sound of mice”) and four videos with low triggering intensity (“scalp massage,” “b-box,” “massage your temples,” and “different triggers”). The participants who were able to experience ASMR (*N* = 32, 65.6% female, Mage = 19.34, *SD* = 0.937, Range [18, 22]) were re-recruited to evaluate the 12 videos. The internal consistency reliability of the first recommended ASMR digital video library was evaluated with Cronbach’s alpha coefficient, which revealed that α = 0.933. In this video collection, “electronic cigarette” and “b-box”; “different triggers,” “eating caviar and sea grape,” and “archeological dig”; “roleplay of energy healing” and “sound of mouse”; “roleplay of energy healing” and “electronic cigarette”; “whispering and personal attention,” “massage your temples,” and “eating caviar and sea grape” showed higher consistency, while “massage your temples” and “ear licking” showed lower consistency.

## Discussion

This study aims to establish the first collection of ASMR-triggering videos that can address the needs of ASMR research, including 60 initial ASMR-triggering videos. In order to allow researchers to have more flexibility and make better choices according to their own needs, we have established the first recommended ASMR-triggering video collection. It includes 12 videos that are categorized into strong, medium and weak according to the intensity and duration of the ASMR triggered.

The most important contribution of this study is the initial screening that established a large ASMR video library. The study initially evaluated the intensity of tingling and the frequency of sustained sensation triggered by 60 ASMR videos. The results showed that the scoring of the ASMR videos on these two dimensions in the experimental group was significantly higher than in the control group, indicating that the video collection can effectively trigger ASMR in the participants who are able to experience ASMR. The strong correlation between these two dimensions of ASMR videos suggests that the higher the intensity of arousal (the intensity of tingling, static-like sensation), the stronger the induced effect. In subsequent ASMR related research, the level of ASMR stimulation can be chosen according to the research purpose to effectively trigger different ASMR experiences. This will provide the foundation for future ASMR related brain research.

Based on this, the first recommended video collection (four videos of three intensity levels: strong, medium, and weak) was established with good reliability (α = 0.933). Future researchers can select the corresponding ASMR video to effectively trigger an ASMR in the participant.

First, considering that the ages of the participants in this study were fairly uniform, and that they had little exposure to ASMR and limited understanding of the stimuli given in the experiment, the level of ASMR could not be tested. In the future, a scale to help screen participants and to describe ASMR experience may be necessary to further assist in the selection of participants for future research. Participants could watch a triggering video and report the tingling frequencies/intensities at different periods of time (e.g., after an interval of a week). With the passage of time, consistent reporting of ASMR responses to the same stimulus will aid in determining if the self-reported ASMR experiences are real, as well as to distinguish between the experimental group and the control group.

Second, the sequential and interactive influence among ASMR videos are difficult to avoid, that is, whether the ASMR experienced by the participant is triggered only by the current stimulus. Research on the encephalic region related to the ASMR experience may provide a solution to this issue in the future.

In the above results, we found that the participants’ tingling and numbing sensation after watching the control video was unexpectedly higher than that of ASMR voice video. Given that there were two videos cutting frozen fruit with knives in the control group video, it was possible that the participants had pain synesthesia when watching the video. Although we did not ask subjects to report synesthesia, interestingly, Barratt and Davis (2015) found in their survey that 5.9% of the ASMR population reported synesthetic experiences, suggesting a possible overlap between the two phenomena. So we think it’s possible that the pain synesthesia that the subjects were producing led them to report higher tingling intensity. In addition, the subject may have the subject effect in the experimental situation, resulting in this unexpected result.

In addition, taking into account that the participants were Chinese and that the videos in the library were taken from YouTube channels in other countries, certain cross-cultural differences might exist. In the ASMR videos with semantic dialogues, we prepared Chinese subtitles so that the participants could understand the conversation. To better present experimental results, in future research, experimenters could set a prompt before each video, to allow participants to understand the content of the next video.

Finally, the ASMR video library in this study has expanded the number and types of videos. However, the selection of materials was mainly based on the subscription numbers of video creators on YouTube and the number of playbacks. There may be triggers to which fewer people are sensitive and through which they can experience ASMR. In the future, with an increase in empirical research on ASMR and the continuous improvement on the part of video creators, the material library should be revised and improved.

## Conclusion

First, this study established an ASMR triggering video library with a total of 60 videos, with an average arousal intensity of 3.96 and a trigger frequency of 3.74 on a 0–8 scale.

Second, for greater convenience of future researchers, we have established the first recommended digital video library with a total of 12 videos, divided into three levels of arousal: strong, medium and weak. Cronbach alpha test showed good reliability and the experiment proved that the videos in the library can effectively trigger ASMR experiences (for scientific research information please contact lmj39270@gmail.com).

## Data Availability Statement

All datasets generated for this study are included in the manuscript/Supplementary Files.

## Ethics Statement

Participants provided written informed consent. This study was approved by the Research Ethics Committee Review of Wenzhou Medical University (Reference number: 2019093).

## Author Contributions

ML and QZ conceived and designed the work, collected, analyzed, and interpreted the data, critically revised the manuscript, and approved the final version of the manuscript to be published. QZ drafted the manuscript.

## Conflict of Interest

The authors declare that the research was conducted in the absence of any commercial or financial relationships that could be construed as a potential conflict of interest.

## Supplementary Material

The Supplementary Videos for this article can be found on the link: https://pan.baidu.com/s/15yFvQoUxMRDPOswJwKy46Q Extract the code: 3xd9

## References

[B1] Barratt E. Spence C. Davis N. (2017). Sensory determinants of the autonomous sensory meridian response (ASMR): understanding the triggers. *PeerJ* 5:e3846. 10.7717/peerj.3846 29018601 PMC5633022

[B2] Barratt E. L. Davis N. J. (2015). Autonomous sensory meridian response (ASMR): a flow-like mental state. *PeerJ* 3:e851. 10.7717/peerj.851 25834771 PMC4380153

[B3] Beverley F. Jim C. Smith S. D. (2017). An examination of personality traits associated with autonomous sensory meridian response (ASMR). *Front. Psychol.* 8:247. 10.3389/fpsyg.2017.00247 28280478 PMC5322228

[B4] Lochte B. C. Guillory S. A. Richard C. A. Kelley W. M. (2018). An fMRI investigation of the neural correlates underlying the autonomous sensory meridian response (ASMR). *Bioimpacts* 8 295–304. 10.15171/bi.2018.32 30397584 PMC6209833

[B5] Peirce J. W. Gray J. R. Simpson S. MacAskill M. R. Höchenberger R. Sogo H. (2019). PsychoPy2: experiments in behavior made easy. *Behav. Res. Methods* 51 195–203. 10.3758/s13428-018-01193-y 30734206 PMC6420413

[B6] Poerio G. Blakey E. Hostler T. Veltri T. (2018). More than a feeling: autonomous sensory meridian response (ASMR) is characterized by reliable changes in affect and physiology. *PLoS One* 13:e0196645. 10.1371/journal.pone.0196645 29924796 PMC6010208

[B7] Smith N. Snider A. M. (2019). ASMR, affect and digitally-mediated intimacy. *Emot. Space Soc.* 30 41–48. 10.1016/j.emospa.2018.11.002

