# Supplementary material for: A Preliminary Compilation of a Digital Video Library on Triggering Autonomous Sensory Meridian Response (ASMR): A Trial Among 807 Chinese College Students
Source: Front Psychol. 2019 Oct 15;10:2274. doi: 10.3389/fpsyg.2019.02274 (PMC6804593; doi:10.3389/fpsyg.2019.02274)
Supplement: Supplementary file 3 [file Table_1.DOCX]

**[Appendix](javascript:;) A**

| Content | YouTuber | Video type | Start time | End time | Duration |
| --- | --- | --- | --- | --- | --- |
| Aromatherapy | Gentle Whispering ASMR | asmr(voice) | 0:03:08 | 0:06:04 | 2:56’06 |
| Tapping and Scratching | Gibi ASMR | asmr(no voice) | 0:18:14 | 0:19:31 | 1:17’04 |
| Eat Chill | Mystic Saurus | asmr(no voice) | 0:02:58 | 0:04:54 | 1:55’15 |
| Facial Cosmetic | Soy ASMR | asmr(no voice) | 0:25:19 | 0:28:13 | 2:53’26 |
| Remove Thorn | Soy ASMR | asmr(no voice) | 0:01:10 | 0:03:04 | 1:54’09 |
| Ear Massage | ASMR darling | asmr(no voice) | 0:25:34 | 0:27:23 | 1:49’20 |
| Touching Your Face | ASMR darling | asmr(no voice) | 0:17:15 | 0:18:35 | 1:20’11 |
| Scalp Massage | ASMR darling | asmr(no voice) | 0:20:19 | 0:21:46 | 1:27’00 |
| Touching Your Face and Mouth Sound | pelegea ASMR | asmr(no voice) | 0:06:05 | 0:08:12 | 2:07’17 |
| Cleaning Ear by Cotton Swab | ASMR PPOMO | asmr(no voice) | 0:25:38 | 0:28:10 | 2:31’11 |
| Eating Caviar and Sea Grape | SAS-ASMR | asmr(no voice) | 0:03:39 | 0:05:53 | 2:14’10 |
| Facial Massage | pelegea ASMR | asmr(voice) | 0:00:04 | 0:03:00 | 2:55’24 |
| Grind Salt | ASMRsurge | asmr(no voice) | 0:14:17 | 0:16:44 | 2:26’25 |
| Cleaning Your Earwax by A Man | YAACHAMA J-ASMR | asmr(no voice) | 0:01:18 | 0:03:49 | 2:31’07 |
| Mixing Beads and Glue | YAACHAMA J-ASMR | asmr(no voice) | 0:00:00 | 0:02:21 | 2:21’00 |
| Brushing Your Ear by A Soft Brush | asmr zeitgrist | asmr(no voice) | 0:24:49 | 0:27:24 | 1:35’03 |
| Mixing Slime Beads | asmr zeitgrist | asmr(no voice) | 0:14:49 | 0:16:09 | 1:19’29 |
| Sound of Soda Water | asmr zeitgrist | asmr(no voice) | 0:05:46 | 0:07:09 | 1:22’06 |
| Ear Massage by A Silicone Beauty Blender | asmr zeitgrist | asmr(no voice) | 0:07:06 | 0:08:04 | 0:57’24 |
| Ear Cleaning by A Swab | asmr zeitgrist | asmr(no voice) | 2:12:25 | 2:13:37 | 1:11’16 |
| Different Trigger | pierreG ASMR | asmr(no voice) | 0:03:30 | 0:04:45 | 1:14’19 |
| Multiple Mouth Sound | Ano ASMR | asmr(no voice) | 0:05:16 | 0:06:42 | 1:26’01 |
| Ear Licking | frivolousFox ASMR | asmr(no voice) | 0:02:54 | 0:05:36 | 2:42’06 |
| Tapping Glass | Nite Shift ASMR | asmr(no voice) | 0:12:38 | 0:14:55 | 2:17’02 |
| Writing | ASMRsurge | asmr(no voice) | 0:00:11 | 0:02:49 | 2:37’46 |
| Soap Carving | ASMRsurge | asmr(no voice) | 0:39:48 | 0:42:46 | 2:57’26 |
| Archaeological Dig Bone | ASMRsurge | asmr(no voice) | 0:06:55 | 0:09:55 | 3:00’00 |
| Mouth Sound | PJ Dreams | asmr(no voice) | 0:01:49 | 0:03:46 | 1:57’08 |
| Tapping A Little Pillow | RaffyTaphyASMR | asmr(voice) | 0:07:45 | 0:10:42 | 2:56’26 |
| Sound of A Scissors | RaffyTaphyASMR | asmr(voice) | 0:06:18 | 0:09:17 | 2:59’13 |
| Tapping A Wood Brick | RaffyTaphyASMR | asmr(voice) | 0:22:39 | 0:25:36 | 2:57’07 |
| Sound of Mouse | Ano ASMR | asmr(voice) | 0:08:03 | 0:09:18 | 1:14’18 |
| Electronic Cigarette | Ano ASMR | asmr(voice) | 0:01:20 | 0:00:02 | 1:09’23 |
| Roleplay of Haircutting | Fred’s Voice ASMR | asmr(voice) | 0:23:26 | 0:25:52 | 2:26’05 |
| B-Box | Seb ASMR | asmr(voice) | 0:00:25 | 0:01:37 | 2:11’13 |
| Scalp Massage by A Man | JoJo’s ASMR | asmr(voice) | 0:04:48 | 0:07:14 | 2:26’12 |
| Multiple Whispering | Massage ASMR | asmr(voice) | 0:11:55 | 0:13:28 | 1:32’27 |
| Massage Someone’s Back | Massage ASMR | asmr(voice) | 0:01:03 | 0:03:35 | 2:31’27 |
| Roleplay of Energy Healing | Massage ASMR | asmr(voice) | 0:00:50 | 0:03:16 | 2:25’27 |
| Sound of Scratching | Massage ASMR | asmr(voice) | 0:04:46 | 0:06:40 | 1:53’26 |
| The Sound of Lg Cracking | Sensor Adi ASMR | asmr(voice) | 0:07:56 | 0:09:50 | 1:53’26 |
| Personal Attention (Male) | ephemeral Rift | asmr(voice) | 0:01:56 | 0:04:11 | 2:14’15 |
| Attempting to Unlock | ephemeral Rift | asmr(voice) | 0:00:00 | 0:01:47 | 1:47’15 |
| Tapping A Wooden Comb | Gentle Whispering ASMR | asmr(voice) | 0:01:02 | 0:03:48 | 2:45’41 |
| Eating Honeycomb | SAS-ASMR | asmr(voice) | 0:00:05 | 0:02:53 | 2:48’07 |
| Cleaning Both Ears at The Same Time | pelage ASMR | asmr(voice) | 0:07:39 | 0:09:11 | 1:32’04 |
| Whispering | Gentle Whispering ASMR | asmr(voice) | 0:14:43 | 0:17:07 | 2:24’16 |
| Whispering and Personal Attention | Gentle Whispering ASMR | asmr(voice) | 0:07:45 | 0:09:31 | 1:46’04 |
| Squeeze Nose Pore | rappeler하쁠리 | asmr(voice) | 0:05:23 | 0:08:46 | 2:42’28 |
| Trigger Words and Ear Cleaning | rappeler하쁠리 | asmr(voice) | 0:00:56 | 0:03:57 | 3:01’12 |
| Roleplay of Makeup | ASMR PPOMO | asmr(voice) | 0:16:46 | 0:19:22 | 2:36’23 |
| Roleplay of Washing Your Hair | ASMR Request | asmr(voice) | 0:02:00 | 0:04:04 | 2:04’20 |
| Roleplay of Taking Off Your Make-Up | Latte ASMR | asmr(voice) | 0:06:17 | 0:09:01 | 2:44’07 |
| Sound of Tongue | chynaunique ASMR | asmr(no voice) | 0:01:43 | 0:03:58 | 2:14’28 |
| Cleaning Your Ear by A Girl | rappeler하쁠리 | asmr(voice) | 0:04:19 | 0:06:37 | 2:18’10 |
| Personal Attention and Relaxing | ASMR darling | asmr(voice) | 0:05:57 | 0:07:40 | 1:43’01 |
| Massage Your Temples | Gentle Whispering ASMR | asmr(voice) | 0:06:26 | 0:09:02 | 3:00’00 |
| Eating Salmon and Octopus | SAS-ASMR | asmr(no voice) | 0:01:01 | 0:03:56 | 2:55’01 |
| Combing Your Hair | ASMR PPOMO | asmr(no voice) | 0:20:23 | 0:20:27 | 2:12’04 |
| Sound of Rain | magic ASMR | asmr(no voice) | 0:40:11 | 0:41:34 | 1:23’16 |
| Cutting Frozen Strawberries | 한세HANSE | control | 0:00:00 | 0:03:00 | 3:00’48 |
| Cutting Frozen Apple | 한세HANSE | control | 0:00:00 | 0:02:58 | 2:58’33 |
| Gem Sugar Made | 한세HANSE | control | 0:00:08 | 0:03:06 | 2:57’05 |
| Dessert Made | 한세HANSE | control | 0:00:08 | 0:02:45 | 2:36’22 |
| Note: information in the table is closed at 2019-4-26 | | | | | |
