# Supplementary material for: A Preliminary Compilation of a Digital Video Library on Triggering Autonomous Sensory Meridian Response (ASMR): A Trial Among 807 Chinese College Students
Source: Front Psychol. 2019 Oct 15;10:2274. doi: 10.3389/fpsyg.2019.02274 (PMC6804593; doi:10.3389/fpsyg.2019.02274)
Supplement: Supplementary file 4 [file Table_2.DOCX]

[**Appendix**](javascript:;) **B**

| Content | Video address | Page view | Subscribe |
| --- | --- | --- | --- |
| Aromatherapy | https://m.youtube.com/watch?v=RVpfHgC3ye0 | 21,304,727 | 1,149,463 |
| Tapping and Scratching | https://m.youtube.com/watch?v=FidhD-izZnk&t=1125s | 11,346,765 | 1,889,073 |
| Eat Chill | https://m.youtube.com/watch?v=SsUIDwyWJ9c&t=241s | 2,135,710 | 174,746 |
| Facial Cosmetic | https://m.youtube.com/watch?v=8sRab7rlwZA&t=41s | 4,729,583 | 489,794 |
| Remove Thorn | https://m.youtube.com/watch?v=HaW4nZ9mc_g&t=357s | 4,407,302 | 489,794 |
| Ear Massage | https://m.youtube.com/watch?v=JeuriGzAnSI&t=1793s | 22,973,171 | 2,325,528 |
| Touching Your Face | https://m.youtube.com/watch?v=DaXwnTk0hUE&t=1248s | 31,543,880 | 2,325,528 |
| Scalp Massage | https://m.youtube.com/watch?v=zltEvQK03Hs | 7,588,317 | 2,325,528 |
| Touching Your Face and Mouth Sound | https://m.youtube.com/watch?v=DXtmlQKIRQA&t=429s | 6,673,205 | 854,600 |
| Cleaning Ear by Cotton Swab | https://m.youtube.com/watch?v=FmpEcgRwq7U&t=1518s | 7,107,424 | 1,779,161 |
| Eating Caviar and Sea Grape | https://m.youtube.com/watch?v=71BsRp4Ehjw&t=133s | 9,772,474 | 6,307,490 |
| Facial Massage | https://m.youtube.com/watch?v=ExjRyEelGaM | 7,921,678 | 854,600 |
| Grind Salt | https://m.youtube.com/watch?v=iTJhMKVKd08&t=1029s | 2,305,629 | 691,239 |
| Cleaning Your Earwax by A Man | https://m.youtube.com/watch?v=LV4FON55e7c&t=1299s | 1,746,651 | 172,162 |
| Mixing Beads and Glue | https://m.youtube.com/watch?v=E8di-PKE5TY&t=1273s | 1,042,356 | 172,162 |
| Brushing Your Ear by A Soft Brush | https://m.youtube.com/watch?v=rn98xM2xfQk&t=1731s | 5,377,575 | 1,093,588 |
| Mixing Slime Beads | https://m.youtube.com/watch?v=rn98xM2xfQk&t=1731s | 5,377,575 | 1,093,588 |
| Sound of Soda Water | https://m.youtube.com/watch?v=rn98xM2xfQk&t=1731s | 5,377,575 | 1,093,588 |
| Ear Massage by A Silicone Beauty Blender | https://m.youtube.com/watch?v=hMJ5MKtIaEY | 5,632,510 | 1,093,588 |
| Ear Cleaning by A Swab | https://m.youtube.com/watch?v=hMJ5MKtIaEY | 5,632,510 | 1,093,588 |
| Different Trigger | https://m.youtube.com/watch?v=xa3foKfR4AA&t=349s | 3,169,914 | 525,650 |
| Multiple Mouth Sound | https://m.youtube.com/watch?v=RyJBEW5-ErA&t=627s | 2,199,557 | 547,469 |
| Ear Licking | https://m.youtube.com/watch?v=7JS9S2GbXyw&t=282s | 8,032,337 | 1,149,463 |
| Tapping Glass | https://m.youtube.com/watch?v=qwqzYZTifcM&t=351s | 1,510,413 | 101,505 |
| Writing | https://m.youtube.com/watch?v=OwuOWfid5IM&t=326s | 5,116,970 | 691,239 |
| Soap Carving | https://m.youtube.com/watch?v=orD6yIilR0A&t=89s | 17,793,435 | 691,239 |
| Archaeological Dig Bone | https://m.youtube.com/watch?v=BGZaBqxqeU0&t=890s | 4,804,027 | 691,239 |
| Mouth Sound | https://m.youtube.com/watch?v=NKM49QayOBI&t=217s | 1,178,432 | 412,691 |
| Tapping A Little Pillow | https://m.youtube.com/watch?v=3x5PzivPJ3Q&t=5s | 14,583,678 | 491,958 |
| Sound of A Scissors | https://m.youtube.com/watch?v=VUj_P0AELoo | 7,442,498 | 491,958 |
| Tapping A Wood Brick | https://m.youtube.com/watch?v=3x5PzivPJ3Q&t=5s | 14,583,678 | 491,958 |
| Sound of Mouse | https://m.youtube.com/watch?v=UxFRHWdx0mw | 3,910,141 | 547,469 |
| Electronic Cigarette | https://www.youtube.com/watch?v=gPZyuxnQvBg | 1,007,351 | 547,469 |
| Roleplay of Haircutting | https://m.youtube.com/watch?v=vRg5mdRDfSg | 564,686 | 364,198 |
| B-Box | https://m.youtube.com/watch?v=b4WscI61998&t=104s | 1,221,919 | 161,863 |
| Scalp Massage by A Man | https://m.youtube.com/watch?v=nheG5trfctw&t=451s | 4,133,844 | 602,790 |
| Multiple Whispering | https://m.youtube.com/watch?v=xY7GC_j_0v8&t=595s | 3,217,058 | 771,255 |
| Massage Someone’s Back | https://m.youtube.com/watch?v=zNCVrO6nlR8&t=260s | 3,931,278 | 771,255 |
| Roleplay of Energy Healing | https://m.youtube.com/watch?v=wIKgLmLphR0&t=99s | 2,349,345 | 771,255 |
| Sound of Scratching | https://m.youtube.com/watch?v=sDKN7ZT5BNk&t=342s | 6,774,307 | 771,255 |
| The Sound of Lg Cracking | https://m.youtube.com/watch?v=sNKDnurDxlk&t=487s | 1,157,307 | 205,197 |
| Personal Attention (Male) | https://m.youtube.com/watch?v=2WDphvKaL7w&t=437s | 9,390,306 | 640,469 |
| Attempting to Unlock | https://m.youtube.com/watch?v=yB_iMNJIB30&t=207s | 3,403,230 | 640,469 |
| Tapping A Wooden Comb | https://m.youtube.com/watch?v=3ErWfiJNQhE | 1,394,529 | 1,149,463 |
| Eating Honeycomb | https://m.youtube.com/watch?v=W3LB4kuEb2c&t=139s | 26,759,630 | 6,307,490 |
| Cleaning Both Ears at The Same Time | https://m.youtube.com/watch?v=C3GIh-FaFw8&t=562s | 5,809,724 | 854,600 |
| Whispering | https://m.youtube.com/watch?v=gs-iqvX6pOA&t=995s | 5,799,264 | 1,149,463 |
| Whispering and Personal Attention | https://www.youtube.com/watch?v=B8jUVci17vE | 17,449,634 | 1,149,463 |
| Squeeze Nose Pore | https://m.youtube.com/watch?v=bQZx5Kv6sfM&t=324s | 3,093,258 | 667,562 |
| Trigger Words and Ear Cleaning | https://m.youtube.com/watch?v=hvSIBY-u5Gw&t=366s | 1,678,152 | 667,562 |
| Roleplay of Makeup | https://m.youtube.com/watch?v=fQCrGw4GCdk&t=722s | 5,116,258 | 1,779,161 |
| Roleplay of Washing Your Hair | https://m.youtube.com/watch?v=x018RC5hJWo&t=342s | 4,419,850 | 1,779,161 |
| Roleplay of Taking Off Your Make-Up | https://m.youtube.com/watch?v=dpGVuHA_vhI&t=535s | 3,145,841 | 740,645 |
| Sound of Tongue | https://m.youtube.com/watch?v=FFa0jjGDO90&t=306s | 2,456,880 | 1,272,676 |
| Cleaning Your Ear by A Girl | https://m.youtube.com/watch?v=ezi6OMZEXCY&t=303s | 5,755,692 | 667,562 |
| Personal Attention and Relaxing | https://m.youtube.com/watch?v=PHu5p8dpxk8&t=424s | 11,143,728 | 2,325,528 |
| Massage Your Temples | https://m.youtube.com/watch?v=RVpfHgC3ye0 | 21,304,727 | 1,149,463 |
| Eating Salmon and Octopus | https://m.youtube.com/watch?v=wsffEFutYQw&t=220s | 22,390,334 | 6,307,490 |
| Combing Your Hair | https://m.youtube.com/watch?v=fpxUn3LH3Io&t=1097s | 7,494,726 | 1,779,161 |
| Sound of Rain | https://m.youtube.com/watch?v=3oxyARXD_RM&t=2542s | 5,271,055 | 1,112,173 |
| Cutting Frozen Strawberries | https://m.youtube.com/watch?v=ZAPhSW6oh1k | 2,438,422 | 1,493,983 |
| Cutting Frozen Apple | https://m.youtube.com/watch?v=ujPq2rum85I | 205,598 | 1,493,983 |
| Gem Sugar Made | https://m.youtube.com/watch?v=7F9jXLpCAPI&t=197s | 1,267,859 | 1,493,983 |
| Dessert Made | https://m.youtube.com/watch?v=oWPhEo27iBM&t=166s | 1,709,912 | 1,493,983 |
| Note: information in the table is closed at 2019-4-26 | | | |
