# Supplementary material for: A Preliminary Compilation of a Digital Video Library on Triggering Autonomous Sensory Meridian Response (ASMR): A Trial Among 807 Chinese College Students
Source: Front Psychol. 2019 Oct 15;10:2274. doi: 10.3389/fpsyg.2019.02274 (PMC6804593; doi:10.3389/fpsyg.2019.02274)
Supplement: Supplementary file 5 [file Table_3.docx]

| [**Appendix**](javascript:;) **C**  The ASMR video stimulus library is tend to trigger descriptive statistics of the intensity and frequency score of the video in ASMR experience | | | | | | |
| --- | --- | --- | --- | --- | --- | --- |
|  | Intense | |  | Frequency | | valid number |
|  | *M* | *SD* |  | *M* | *SD* |  |
| Eating caviar and sea grape | 5.33 | 3.20 |  | 6.60 | 3.02 | 15 |
| Grind salt | 5.20 | 3.03 |  | 4.40 | 2.61 | 5 |
| Cleaning your ear by a girl | 5.17 | 2.69 |  | 5.00 | 2.89 | 12 |
| Ear licking | 5.00 | 2.81 |  | 5.29 | 2.85 | 17 |
| Electronic cigarette | 5.00 | 3.65 |  | 4.75 | 3.50 | 4 |
| Sound of soda water | 4.87 | 2.92 |  | 4.80 | 2.78 | 15 |
| Roleplay of makeup | 4.87 | 2.00 |  | 4.53 | 2.26 | 15 |
| Ear massage | 4.82 | 1.83 |  | 4.73 | 2.57 | 11 |
| Cleaning your earwax by a man | 4.75 | 2.49 |  | 5.08 | 2.61 | 12 |
| Cleaning ear by Cotton swab | 4.70 | 2.68 |  | 5.35 | 2.62 | 20 |
| Trigger words and ear cleaning | 4.67 | 2.87 |  | 4.33 | 2.69 | 9 |
| Tapping glass | 4.65 | 3.45 |  | 4.50 | 3.47 | 20 |
| Scalp massage by a man | 4.58 | 3.00 |  | 4.33 | 2.84 | 12 |
| Personal attention （male） | 4.40 | 2.30 |  | 4.00 | 1.58 | 5 |
| Eating Salmon and octopus | 4.33 | 3.03 |  | 4.07 | 2.75 | 27 |
| Multiple mouth sound | 4.33 | 2.74 |  | 3.53 | 2.45 | 15 |
| Brushing your ear by a soft brush | 4.15 | 2.91 |  | 4.30 | 2.94 | 20 |
| Cleaning both ears at the same time | 4.09 | 2.66 |  | 4.55 | 2.95 | 11 |
| Tapping a wood brick | 4.07 | 2.76 |  | 3.80 | 2.57 | 15 |
| Facial massage | 4.06 | 3.03 |  | 4.41 | 2.67 | 17 |
| Ear cleaning by a swab | 3.96 | 2.41 |  | 4.19 | 2.54 | 27 |
| Tapping a little pillow | 3.96 | 2.52 |  | 3.67 | 2.39 | 27 |
| The sound of LG cracking | 3.95 | 2.95 |  | 3.85 | 2.76 | 20 |
| Writing | 3.94 | 2.90 |  | 3.65 | 3.02 | 17 |
| Squeeze nose pore | 3.89 | 2.78 |  | 3.93 | 2.63 | 27 |
| Sound of a scissors | 3.88 | 2.62 |  | 3.71 | 2.44 | 17 |
| Facial cosmetic | 3.83 | 2.59 |  | 3.33 | 2.88 | 6 |
| Mouth sound | 3.83 | 3.10 |  | 3.67 | 2.87 | 12 |
| Roleplay of haircutting | 3.82 | 2.04 |  | 3.64 | 2.16 | 11 |
| Eat chill | 3.75 | 2.22 |  | 3.75 | 2.22 | 4 |
| Touching your face and mouth sound | 3.65 | 2.52 |  | 4.47 | 3.36 | 17 |
| Roleplay of taking off your make-up | 3.65 | 2.80 |  | 4.18 | 3.07 | 17 |
| Whispering | 3.64 | 3.32 |  | 3.64 | 3.44 | 11 |
| Sound of scratching | 3.61 | 2.93 |  | 4.06 | 3.02 | 18 |
| Sound of mouse | 3.56 | 2.35 |  | 4.00 | 2.69 | 9 |
| Archaeological dig bone | 3.45 | 1.92 |  | 3.55 | 2.25 | 11 |
| Roleplay of washing your hair | 3.35 | 2.77 |  | 3.40 | 2.50 | 20 |
| Tapping and scratching | 3.33 | 1.87 |  | 3.56 | 2.56 | 9 |
| Sound of tongue | 3.29 | 2.47 |  | 3.24 | 2.86 | 17 |
| Eating honeycomb | 3.26 | 2.88 |  | 3.74 | 2.90 | 19 |
| Soap carving | 3.25 | 1.76 |  | 3.92 | 2.43 | 12 |
| Combing your hair | 3.24 | 2.31 |  | 3.59 | 2.98 | 17 |
| Remove thorn | 3.18 | 1.66 |  | 3.64 | 2.69 | 11 |
| Whispering and personal attention | 3.15 | 2.20 |  | 3.07 | 2.30 | 27 |
| Roleplay of energy healing | 3.13 | 2.36 |  | 3.07 | 2.31 | 15 |
| Mixing beads and glue | 3.11 | 2.20 |  | 3.33 | 2.55 | 9 |
| Attempting to unlock | 3.00 | 2.10 |  | 2.36 | 1.80 | 11 |
| Sound of rain | 3.00 | 2.26 |  | 2.75 | 1.55 | 12 |
| Ear massage by a Silicone beauty blender | 2.83 | 2.09 |  | 2.94 | 2.51 | 18 |
| Mixing slime beads | 2.75 | 1.82 |  | 2.92 | 2.35 | 12 |
| Multiple whispering | 2.71 | 2.44 |  | 2.65 | 2.42 | 17 |
| Personal attention and relaxing | 2.60 | 1.81 |  | 2.20 | 1.30 | 5 |
| Aromatherapy | 2.59 | 2.00 |  | 2.47 | 1.70 | 17 |
| Different trigger | 2.44 | 1.51 |  | 2.22 | 1.20 | 9 |
| Massage your temples | 2.42 | 2.11 |  | 2.58 | 1.83 | 12 |
| Touching your face | 2.37 | 2.02 |  | 2.78 | 2.55 | 27 |
| Scalp massage | 2.33 | 1.56 |  | 2.83 | 2.08 | 12 |
| B-box | 2.33 | 1.56 |  | 2.50 | 1.73 | 12 |
| Massage someone's back | 2.33 | 1.56 |  | 2.92 | 2.35 | 12 |
| Tapping a wooden comb | 2.20 | 1.48 |  | 1.80 | 1.32 | 10 |
