# Supplementary material for: A Preliminary Compilation of a Digital Video Library on Triggering Autonomous Sensory Meridian Response (ASMR): A Trial Among 807 Chinese College Students
Source: Front Psychol. 2019 Oct 15;10:2274. doi: 10.3389/fpsyg.2019.02274 (PMC6804593; doi:10.3389/fpsyg.2019.02274)
Supplement: Supplementary file 6 [file Table_4.doc]

**The captions for the videos**

Below is a brief description of the content of each video fragment. For more detailed video content and parameters, please refer to the appendix.

brief description:

| 1．Aromatherapy | Supplementary Video 1 shows “a woman treats you with aromatherapy, whispers gently in your ear, and surround yourself with scent to make you feel relaxed”. |
| --- | --- |
| 2.tapping and scratching | Supplementary Video 2 shows “a woman scratched a bag gently and tap it, and made some sound”. |
| 3.eat chill | Supplementary Video 3 shows “a woman ate Fried green pepper in front of the camera, comfortable chewing sound” |
| 4.facial cosmetic | Supplementary Video 4 shows “a woman lie down comfortably and give her a gentle face massage” |
| 5.remove thorn | Supplementary Video 5 shows “the microphone is covered with needles, which are removed with tweezers”. |
| 6.ear massage | Supplementary Video 6 shows “a role play: a woman massages your ears with oil”. |
| 7.touching your face | Supplementary Video 7 shows “a woman constantly touches the camera as if she were touching your face”. |
| 8.Scalp massage | Supplementary Video 8 shows “a role play: a woman massages your scalp”. |
| 9.touching your face and mouth sound | Supplementary Video 9 shows “a woman constantly touches the camera as if she were touching your face, and make a soft sound”. |
| 10.cleaning ear by Cotton swab | Supplementary Video 10 shows “cleaning the ear on the 3D microphone with a cotton swab”. |
| 11.eating caviar and sea grape | Supplementary Video 11 shows “a woman eats sea grapes and caviar on camera and make some chewing sound”. |
| 12.facial massage | Supplementary Video 12 shows “a role play: a woman massages your face with baby oil”. |
| 13.grind salt | Supplementary Video 13 shows “grinding the salt slowly and mechanically in front of the camera”. |
| 14.cleaning your earwax by a man | Supplementary Video 14 shows “using an ear scoop to remove the ear from the 3D microphone, as if cleaning earwax”. |
| 15.mixing beads and glue | Supplementary Video 15 shows “placing the glue and the clear beads in a large bowl and slowly mix them together with a blender”. |
| 16.brushing your ear by a soft brush | Supplementary Video 16 shows “using a soft brush to gently brush the ears of the 3D microphone”. |
| 17.mixing slime beads | Supplementary Video 17 shows “placing the 3D microphone on slime and slowly scratching slime nearby your ears”. |
| 18.sound of soda water | Supplementary Video 18 shows “placing two small cups of soda water next to the ears of the 3D microphone”. |
| 19.ear massage by a Silicone beauty blender | Supplementary Video 19 shows “massaging the ears of 3D with silicone makeup eggs microphone as if massaging your ears”. |
| 20.ear cleaning by a swab | Supplementary Video 20 shows “a man cleans the ear on the 3D microphone with a cotton swab”. |
| 21.different trigger | Supplementary Video 21 shows “a man makes all kinds of interesting trigger sounds in front of the camera”. |
| 22.multiple mouth sound | Supplementary Video 22 shows “use the mouth to make various sounds around the microphone”. |
| 23.ear licking | Supplementary Video 23 shows “a woman licks the ear of a 3D microphone gently as if licks your ears” |
| 24.tapping glass | Supplementary Video 24 shows “a man taps gently on various bottles filled with water and makes some comfortable sounds”. |
| 25.writing | Supplementary Video 25 shows “writing with a pen on a piece of white paper slowly 0in a close-up shot”. |
| 26.soap carving | Supplementary Video 26 shows “a man is carving a cartoon character out of a bar of soap”. |
| 27.Archaeological dig bone | Supplementary Video 27 shows “simulate the process of archaeological investigation with archaeological toys”. |
| 28.mouth sound | Supplementary Video 28 shows “a man is making various sounds with his mouth around a microphone”. |
| 29.tapping a little pillow | Supplementary Video 29 shows “a man is tapping a small white pillow gently and with rhythm”. |
| 30.sound of a scissors | Supplementary Video 30 shows “use a variety of scissors to make clipping sounds around the microphone”. |
| 31.tapping a wood brick | Supplementary Video 31 shows “ a man is using two small pieces of wood to make all kinds of clashing sounds”. |
| 32.sound of mouse | Supplementary Video 32 shows “Stop-motion animation mouse in the left and right side of the voice”. |
| 33.Electronic cigarette | Supplementary Video 33 shows “the sound of a man smoking an e-cigarette around a microphone”. |
| 34.roleplay of haircutting | Supplementary Video 34 shows “a role play: a man cuts your hair like a barber in a shop”. |
| 35.b-box | Supplementary Video 35 shows “gently b-boxing”. |
| 36.Scalp massage by a man | Supplementary Video 36 shows “slowly touch the dust cover on the microphone as if it were massaging your scalp”. |
| 37.multiple whispering | Supplementary Video 37 shows “whisper softly in your ear”. |
| 38.massage someone's back | Supplementary Video 38 shows “a man showed how to massages men’s back by massaging a woman’s back”. |
| 39.roleplay of energy healing | Supplementary Video 39 shows “a man ACTS as your energy therapist and helps you to recover your energy”. |
| 40.sound of scratching | Supplementary Video 40 shows “a man swept the paper over the microphone and made some sounds”. |
| 41.The sound of LG cracking | Supplementary Video 41 shows “the sound of crushing the protective film of a mobile phone”. |
| 42.personal attention （male） | Supplementary Video 42 shows “a man has a personal attention conversation with you”. |
| 43.attempting to unlock | Supplementary Video 43 shows “the author takes the audience to try to open the password lock process”. |
| 44.tapping a wooden comb | Supplementary Video 44 shows “a woman tapped a wooden comb around the microphone”. |
| 45.eating honey | Supplementary Video 45 shows “an Asian girl eats the wild honey, and make some chewing sound”. |
| 46.Cleaning both ears at the same time | Supplementary Video 46 shows “the twins cleaned the ears of the 3D microphone as if cleaned your ears”. |
| 47.whispering | Supplementary Video 47 shows “a woman was whispering into the microphone gentlely to make you feel relaxed”. |
| 48.whispering and personal attention | Supplementary Video 48 shows “a woman at the microphone brushed her hair and whispered”. |
| 49.Squeeze nose pore | Supplementary Video 49 shows “use a silicone toy to simulate the black head out of pores”. |
| 50.trigger words and ear cleaning | Supplementary Video 50 shows “a woman cleans the ear on a 3D microphone and says the trigger word”. |
| 51.roleplay of makeup | Supplementary Video 51 shows “a role play: An Asian girl will help you with your makeup”. |
| 52.roleplay of washing your hair | Supplementary Video 52 shows “a role play : A European girl washes your hair”. |
| 53.roleplay of taking off your make-up | Supplementary Video 53 shows “a role play : An Asian girl takes off your makeup”. |
| 54.sound of tongue | Supplementary Video 54 shows “a black girl makes different mouth sounds around the 3D microphone”. |
| 55.cleaning your ear by a girl | Supplementary Video 55 shows “a woman cleaning a 3D microphone is like cleaning your ears”. |
| 56.personal attention and relaxing | Supplementary Video 56 shows “gently brush the screen with a brush as if it were brushing your face and engage in a personal attention conversation”. |
| 57.massage your temples | Supplementary Video 57 shows “a woman simulates massaging your temples to make you feel relaxed”. |
| 58.eating Salmon and octopus | Supplementary Video 58 shows “a woman ate a large piece of salmon, making occasional chewing sounds”. |
| 59.combing your hair | Supplementary Video 59 shows “in video, a woman is helping another person brush her hair gently”. |
| 60.sound of rain | Supplementary Video 60 shows “the rain fell on the transparent umbrella against a black background, making a sound”. |
| 61.Cutting frozen strawberries | Supplementary Video 61 shows “use ceramic knife to cut frozen strawberries slowly and mechanically”. |
| 62.Cutting frozen apple | Supplementary Video 62 shows “use ceramic knife to cut frozen apples slowly and mechanically”. |
| 63.Gem sugar made | Supplementary Video 63 shows “the process of making gemstone sugar is shown in detail ”. |
| 64.Dessert made | Supplementary Video 64 shows “the process of making a dessert is shown in detail”. |

|  |
| --- |
